# Early assessment of China’s 2015 tobacco tax increase

**DOI:** 10.2471/BLT.17.205989

**Published:** 2018-05-14

**Authors:** Mark Goodchild, Rong Zheng

**Affiliations:** a Department for the Prevention of Noncommunicable Diseases, World Health Organization, avenue Appia 20, 1211 Geneva 27, Switzerland.; b School of International Trade and Economics, University of International Business and Economics, Beijing, China.

## Abstract

In 2015, the Chinese government raised tobacco excise tax for the first time since 2009. Changing from previous practice, the State Tobacco Monopoly Administration raised its cigarette prices at the same time. We assessed the early impact of the 2015 tax increase on cigarette prices, sales volumes, tax revenue generation and the potential effect on prevalence of smoking in China. Between 2014 and 2016, the retail price of cigarettes increased on average by 11%, with the cheapest category of cigarette brands increasing by 20%. The average proportion of tax in the price of cigarettes rose from 51.7% to 55.7%. Annual cigarette sales decreased by 7.8%, from 127 to 117 billion packs. The increase in cigarette prices could be associated with a 0.2% to 0.6% decrease in the proportion of adults smoking, representing between 2.2 and 6.5 million fewer smokers. Tax revenues from cigarettes increased by 14%, from 740 to 842 billion Chinese yuan between 2014 and 2016, reflecting an extra 101 billion Chinese yuan in tax revenues for the government. The 2015 tax increase shows that tobacco taxation can provide measurable benefits to both public health and finance in China. The experience also highlights the potential for tobacco taxation to contribute to China’s broader development targets, including the sustainable development goals and Healthy China 2030. Looking forward, this link to development can be facilitated through multisectoral research and dialogue to develop consistent cross-sectoral objectives for tobacco tax policy design and implementation.

## Résumé

En 2015, le gouvernement chinois a augmenté la fiscalité sur le tabac pour la première fois depuis 2009. Contrairement aux pratiques passées, l’Administration du monopole d’État sur le tabac a décidé d’augmenter en même temps les prix des cigarettes. Nous avons évalué les premiers impacts de la hausse de 2015 des taxes sur le tabac, les volumes vendus, les recettes fiscales qui en ont découlé et l’effet potentiel de cette mesure sur la prévalence du tabagisme en Chine. Entre 2014 et 2016, le prix de vente au détail des cigarettes a augmenté de 11% en moyenne, avec une augmentation de 20% pour les marques les moins chères. La proportion moyenne des taxes sur le prix des cigarettes est passée de 51,7% à 55,7%. Les ventes annuelles de cigarettes ont baissé de 7,8%, passant de 127 à 117 milliards de paquets. L’augmentation des prix des cigarettes pourrait être associée à une réduction comprise entre 0,2% et 0,6% de la proportion de fumeurs dans la population adulte, ce qui représente entre 2,2 et 6,5 millions de fumeurs en moins. Les recettes fiscales obtenues sur la vente de cigarettes ont augmenté de 14% (740 milliards de yuans en 2014 contre 842 milliards en 2016), apportant ainsi au gouvernement 101 milliards de yuans de recettes fiscales supplémentaires. La hausse de la fiscalité opérée en 2015 en Chine montre que la taxation du tabac peut avoir des bénéfices appréciables à la fois sur la santé publique et sur les finances publiques. Cette expérience révèle aussi le potentiel de la taxation du tabac dans la poursuite des objectifs plus larges de développement de la Chine, notamment des objectifs de développement durable et des objectifs du programme «Healthy China 2030» (une Chine saine en 2030). À l’avenir, ce lien avec les objectifs de développement pourrait être optimisé par des recherches et un dialogue multisectoriels visant à définir des objectifs intersectoriels cohérents pour l’élaboration et l’application des politiques fiscales sur le tabac.

## Resumen

En 2015, el gobierno chino subió el impuesto especial sobre el tabaco por primera vez desde 2009. De manera distinta a la práctica anterior, la Administración Estatal de Monopolio de Tabaco subió los precios de los cigarrillos al mismo tiempo. Se evalúa el impacto inicial de la subida de impuestos de 2015 sobre los precios de los cigarrillos, los volúmenes de venta, la generación de ingresos tributarios y el posible efecto sobre la prevalencia del tabaco en China. Entre 2014 y 2016, el precio minorista de los cigarrillos subió de media un 11 % y la categoría más barata de marcas de cigarrillos subió en un 20 %. La proporción media de impuestos en el precio de los cigarrillos subió del 51,7 % al 55,7 %. Las ventas anuales de cigarrillos cayeron un 7,8 %, de 127 000 a 117 000 millones de paquetes. La subida de los precios de los cigarrillos podría estar asociada con una reducción del 0,2 % al 0,6 % en la proporción de fumadores adultos, lo que representa entre 2,2 y 6,5 millones de fumadores menos. Los ingresos fiscales procedentes de los cigarrillos aumentaron un 14%, de 740 000 a 842 000 millones de yuanes chinos entre 2014 y 2016, lo que se refleja un aumento de 101 000 millones de yuanes chinos en ingresos fiscales para el gobierno. La subida tributaria de 2015 muestra que el impuesto sobre el tabaco puede brindar unos beneficios considerables tanto para la salud pública como para las finanzas en China. Los ingresos tributarios de los cigarrillos subieron un 14 %, de 740 000 a 842 000 millones de yuanes chinos entre 2014 y 2016, lo que equivale a 15 000 millones adicionales de dólares estadounidenses en ingresos fiscales para el gobierno. La subida de los impuestos sobre el tabaco en China en 2015 supone una demostración de que los impuestos sobre el tabaco podrían generar beneficios cuantificables tanto para la salud pública como para las finanzas. La experiencia también destaca el potencial de los impuestos sobre el tabaco de contribuir a los objetivos de desarrollo más amplios de China, incluidos los objetivos de desarrollo sostenible y salud de China para 2030. De cara al futuro, este vínculo con el desarrollo puede facilitarse a través de la investigación y el diálogo multisectoriales para desarrollar objetivos intersectoriales coherentes para el diseño y la implementación de la política tributaria del tabaco.

## ملخص

في عام 2015، قامت الحكومة الصينية برفع الضريبة غير المباشرة على التبغ للمرة الأولى منذ عام 2009. وتحولًا عن الممارسة السابقة فقد قامت إدارة حصر التبغ الوطنية برفع أسعار السجائر في نفس الوقت. وقد قمنا بتقييم التأثير المبكر للزيادة الضريبية لعام 2015 على أسعار السجائر وحجم المبيعات وإدرار إيرادات الضرائب والتأثير المحتمل لانتشار التدخين في الصين. وقد ارتفع سعر البيع بالتجزئة للسجائر بمتوسط 11% ما بين عامي 2014 و2016، مع ارتفاع أسعار أرخص الأصناف من ماركات السجائر بنسبة 20%. وارتفع متوسط نسبة الضريبة في سعر السجائر من 51.7% إلى 55.7%. وانخفضت مبيعات السجائر السنوية بنسبة 7.8% وذلك من 127 إلى 117 مليار علبة سجائر. ويمكن أن يرتبط الارتفاع في أسعار السجائر بالانخفاض بنسبة 0.2% إلى 0.6% في نسبة تدخين البالغين، مما يمثل انخفاض بمعدل ما بين 2.2 و6.5 مليون في أعداد المدخنين. وقد ارتفعت إيرادات الضرائب من السجائر بنسبة 14% وذلك من 740 مليار يوان صيني إلى 842 مليار يوان صيني ما بين عامي 2014 و2016، وهو ما يعادل 101 مليار يوان صيني إضافي في إيرادات الضرائب الحكومية. وتوضح زيادة الضريبة في عام 2015 الدليل على أنه من شأن فرض الضرائب على التبغ أن يقدم فوائد ملموسة لكل من الصحة العامة والتمويل في الصين. وتسلط التجربة كذلك الضوء على إمكانية إسهام الضرائب المفروضة على التبغ في تحقيق أهداف التنمية الأوسع نطاقًا في الصين، بما يشمل أهداف التنمية المستدامة وهدف الحصول على صين بصحة جيدة عام 2030. وبالتطلع للمستقبل سنجد أنه من الممكن تيسير هذه الصلة بالتنمية من خلال البحوث متعددة القطاعات والحوار لتطوير أهداف متناسقة ومشتركة بين عدة قطاعات لتصميم سياسة ضريبة التبغ وتنفيذها

## 摘要

2015 年，中国政府自 2009 年以来首次上调烟草消费税。与2009年卷烟消费税调整不同的是，国家烟草专卖局此次同时提高了卷烟价格。我们评估了 2015 年税收上调对卷烟价格、销量、税收收入的早期影响以及对中国吸烟流行率的潜在影响。2014 年至 2016 年，卷烟零售价格平均上涨了11%，最便宜的卷烟品类的价格上涨了20%；税收占零售价格的比重平均从 51.7% 增加至 55.7%；年度卷烟销量从 1270 亿包下降至 1170 亿包，降幅为 7.8%；卷烟价格上涨预计使得成人吸烟率下降 0.2% 至 0.6%，这意味着吸烟者人数减少220 万至650万。2014至 2016年，烟草税收收入从7400 亿人民币增加至 8420 亿人民币，涨幅为14%。对政府而言，这相当于增加约1010亿人民币的额外税收收入。中国 2015 年上调烟草税的举措证明提高烟草税是对公众健康和政府收入均有益处的“双赢”策略。这次税改同时也证明烟草税 具备促进中国实现更广阔的发展目标的潜力，包括可持续发展目标和“健康中国 2030”规划纲要的目标。展望未来，可通过加强跨学科研究和围绕跨部门共同目标的对话来完善中国烟草税的制度设计，以促进可持续发展目标的实现。

## Резюме

В 2015 году, впервые с 2009 года, правительство Китая повысило акцизный налог на табак. В отличие от ранее принятой практики, Государственное табачное монопольное управление одновременно повысило и цены на сигареты. Авторы оценили первые последствия повышения налога на стоимость сигарет в 2015 году, объемы продаж, объемы налоговой выручки и потенциальное воздействие этого фактора на частоту курения в Китае. В период между 2014 и 2016 годами розничные цены на сигареты выросли в среднем на 11%, при этом наиболее дешевые марки подорожали на 20%. Средняя доля налогов в стоимости сигарет выросла с 51,7 до 55,7%. Годовой объем продаж сигарет снизился на 7,8% — с 127 до 117 миллиардов пачек. Рост цен на сигареты может ассоциироваться с падением доли взрослых курильщиков на 0,2–0,6%. Это означает, что таких курильщиков стало меньше на 2,2–6,5 миллиона человек. Налоговая выручка от продажи сигарет выросла в период между 2014 и 2016 годами на 14% — с 740 до 842 миллиардов юаней. Это означает, что правительство получило около 101 миллиарда юаней прибыли в виде налогов. Повышение налога на табак в 2015 году в Китае демонстрирует, что налогообложение табака может приносить заметную пользу как финансовому сектору, так и здравоохранению. Этот опыт также указывает на то, что налогообложение табака может быть полезным и для достижения более широких целей развития, которые перед собой ставит Китай, включая устойчивое развитие и «здоровый Китай к 2030 году». Если смотреть в будущее, то достичь этой связи с развитием можно через разнонаправленные исследования и диалог между различными секторами, чтобы выработать устойчивые кросс-секторальные цели для последующей разработки политики налогообложения табака и ее реализации.

## Introduction

In China, tobacco use is contributing to the increase in noncommunicable diseases.^1^ More than 1 million Chinese adults die annually from tobacco use and this number is estimated to increase in coming years.^2^^,^^3^ In 2012, the United Nations (UN) addressed heightened concerns about the impact of noncommunicable diseases in a High-Level Meeting of the UN General Assembly. The Declaration from this meeting notes the “increased burden that noncommunicable diseases impose through impoverishment from long-term treatment costs, and from productivity losses that threaten household incomes and the economies of Member States.”^4^ Indeed, diseases caused by smoking account for around 3% of health expenditures in China, while out-of-pocket medical expenditures, due to smoking, impoverish more than 10 million Chinese households each year.^5^^,^^6^

In 2015, the UN adopted *Transforming our world: the 2030 agenda for sustainable development*,^7^ which includes 17 goals that countries have agreed to achieve. Sustainable development goal (SDG) 3, that is, “ensure healthy lives and promoting well-being for all ages”, includes target 3.4 to reduce by one third premature mortality from noncommunicable diseases by 2030.^7^

SDG 3 also includes target 3.a to strengthen country implementation of the World Health Organization (WHO) Framework Convention on Tobacco Control (FCTC).^7^^,^^8^ China was an early adopter of the FCTC having ratified the Treaty in 2005. Since ratification, tobacco control efforts in China have accelerated with interventions including tighter controls on tobacco marketing, improved health warnings and bans on smoking in public places in several cities.

Tobacco taxation is a cornerstone of global tobacco control efforts, with Article 6 of the FCTC recognizing tax as an important and effective means of reducing the demand for tobacco. Taxation is recognized to be the single most effective tobacco control measure available and the guidelines for Article 6 implementation emphasizes that any comprehensive tobacco control strategy should include taxation.^8^^,^^9^

In 2015, China introduced its fourth major national tobacco tax reform since 1994. Many other large developing countries like Brazil, the Philippines, South Africa and Ukraine have raised tobacco taxes to help meet tobacco control objectives.^10^ In Brazil, higher tobacco taxes contributed to almost half of the decrease in the proportion of adults smoking, from 34.4% in 1989 to 14.7% in 2013.^11^^,^^12^ Similarly, in the Philippines, the so-called sin-tax reforms that began in 2012 was associated with a decrease in the proportion of adults smoking, from 27.9% in 2009 to 22.5% in 2015.^13^

Retrospective studies have shown the importance of tobacco taxation to public health outcomes. In the United States of America, for example, a study found that a 10% increase in cigarette taxes would decrease the number of deaths from respiratory cancers by 1.5%.^14^ The French government has increased cigarette taxes substantially from the mid-1990s, with cigarette prices tripling in real terms by 2005. Among French males, rates of adult lung cancer deaths fell by 50% over the same period.^15^^,^^16^

Here we assess the immediate impact of the 2015 Chinese tobacco tax increase on cigarette prices, sales volumes and tax revenues across the different price categories of China’s cigarette market. The study also explores the potential impact on smoking prevalence and considers the way forward for tax policy design in China.

## Tax reform and cigarette pricing

Table 1 shows the excise system for cigarettes in China, before and after the tax increase in May 2015. The tax increase occurred at the wholesale level, with a specific excise of ¥ 0.10/pack being introduced together with a 6% increase in the existing *ad valorem* tax. Other indirect taxes on cigarettes, which remained unchanged, include value added tax (VAT) at 17%; an urban maintenance and construction tax and education surcharge (known as the C&E tax) of 12% applied on excise and VAT revenue; and a tobacco leaf tax of 20% on the value of leaf production. The definition of total tax on cigarettes in this article includes excise and these other indirect taxes, but excludes China’s enterprise income tax. This exclusion is to be consistent with WHO’s practice of excluding corporate income tax from the measure of taxes faced by the consumer.^10^

**Table 1:** Cigarette classification and excise tax structure in China, 2015

Level, type of excise	Producer price range, ¥/pack^a^	Tax	
		Before 10 May 2015	From 10 May 2015
**Producer**			
*Specific for all classes*	> 0	0.06 ¥ /pack	0.06 ¥/pack
*Ad valorem tax^b^*			
Class I	> 10	56%	56%
Class II	7–10	56%	56%
Class III	3–7	36%	36%
Class IV	1.65–3	36%	36%
Class V	< 1.65	36%	36%
**Wholesale**			
*Specific for all classes*	> 0	No tax	0.10 ¥ pack
*Ad valorem tax for all classes^b^*	> 0	5%	11%

¥: yuan.

^a^ One cigarette pack contains 20 sticks.

^b^ The *ad valorem* excise rates are applied to the respective producer and wholesale price of each class.

Note: The conversion rate in 2016 was 1 United States dollar to  ¥ 6.64.

The 2015 tobacco tax increase coincided with an announcement from the State Tobacco Monopoly Administration that the wholesale price of all cigarette brands will increase by 6%. The monopoly is a state-owned monopoly that controls many aspects of China’s tobacco industry, including the price and profit margins of cigarette producers, wholesalers and independent retailers. The monopoly, therefore, plays a pivotal role in determining how any tax increase is transmitted to the consumer via changes in price. The announcement was an important change from previous practice, because the monopoly did not increase cigarette prices following the last tobacco tax increase in 2009.^17^^,^^18^ Thus, the 2015 tax reform was associated with some of new the tax burden being passed along to consumers. In addition, the monopoly directed its provincial branches to set retail prices to retain retail margins of at least 10% and they circulated a nationwide bulletin listing the advised retail price of all cigarettes.^19^

Economic theory suggests that a monopolist will tend to increase retail prices by more than the tax increase, so-called over-shift.^20^ Since 2010, however, the monopoly has been implementing an optimization marketing strategy, requiring its cigarette manufacturers to focus on key brands that can compete with international brands, while maintaining cheap offerings in the market. This strategy serves to encourage smoking by poor people and uptake among new smokers. The monopoly has therefore increased the price of individual brands very infrequently, which in turn has contributed to an increase in cigarette affordability and consumption over time.^21^ Thus, over-shifting of taxes may not be consistent with the monopoly’s strategy, particularly with respect to the availability of cheap cigarettes.

## Early outcome assessment

We used the WHO Tobacco Tax Simulation model to assess the impact of China’s 2015 tax increase by each price category.^22^^,^^23^ The model is originally a forecasting tool, but here we populated the model with actual price and sales volume data for the years 2014, 2015 and 2016. We obtained this data from the tobacco monopoly’s annual bulletins.^24^ One advantage of this model is that it details tax and price changes at different levels along the supply-chain, namely producers, wholesalers and retailers. This is relevant, because the government levies different taxes on these agents and applies a two-tiered excise system on producers.

The monopoly produces around 89 brand families with each of these families offering multiple variations in terms of price, packaging and quality. In 2015, there were some 870 different brands on the market, with retail prices ranging from 2.5–100.0 Chinese yuan (¥) per pack (equivalent to 0.4–16.1 United States dollars, US$). The monopoly categorizes the brands into five fixed price categories, from the most expensive brands in class I to the cheapest brands in class V, based on their producer price, known in China as the allocation price (Table 1).

We selected five brands to be representative of each price category with these brands having a large market share within their respective class. Since class I has a wide retail price span, we divided class I in to (A) and (B), where I(A) spans prices above ¥ 43/pack. To calculate the tax yield (i.e. tax per pack) and price in each class, we entered the allocation, wholesale and retail prices of these 6 brands into the tobacco tax simulation model. We calculated weighted average tax yields and prices for the entire market, using the market share of each class on a sales volume basis. 

From the monopoly records, we obtained the total tax revenues for the entire cigarette market in 2014, 2015, and 2016.^24^^,^^25^ However, as tax revenues by class were not available, we estimated the tax revenues for each class from the tobacco tax simulation model, then re-scaled them to match the monopoly’s aggregate records in each year. Note the tax revenues predicted by the model in this manner were within 5% of the tax revenues reported by the monopoly. Therefore, any corrections were minor.

### Cigarette consumption

In China, retail sales are a good indicator of cigarette consumption, because the domestic illicit market is small.^26^ However, other factors can confound our ability to test the sensitivity of consumption to the 2015 tax increase. For example, the timeframe coincides with bans on smoking in public places in Beijing and several other cities, slower economic growth compared to earlier decades, as well as the residual effect of China’s anti-extravagance campaign.^26^ Also household survey data is lacking to quantify any impact on the proportion of smokers among China’s population after the 2015 tax increase. Consequently, this study does not attempt to measure the elasticity of demand for cigarettes.

Instead, we used evidence from the literature to explore the potential impact of the 2015 tax increase on smoking. For example, recent studies have found that the price elasticity of demand for cigarettes in China is around −0.5.^27^^,^^28^ This is similar to estimates from other developing countries, meaning that a 10% increase in the price of cigarettes will reduce cigarette consumption by 5%.^29^ This price elasticity reflects a combination of conditional demand, i.e. the amount consumed by smokers and number of people smoking. Evidence shows that about half of the reduction in cigarettes sales due to price increase is because people quit smoking and the remainder is a decrease in the amount of cigarettes consumed by continuing smokers.^29^ Hence, the price prevalence elasticity of demand in developing countries is expected to be about −0.25.

Several studies have modelled the expected impact of cigarette tax increases by applying price prevalence elasticities of −0.1, −0.2 and −0.3 for high-, middle- and low-income countries respectively.^23^^,^^30^^,^^31^ In this paper, we explore the potential impact of the 2015 tax increase on smoking prevalence by applying an elasticity range of between −0.1 and −0.3. That is, a 10% increase in the price of cigarettes will reduce smoking prevalence by 1–3% under the caveat that cigarettes do not become more affordable over time.

### Early findings

#### Cigarette prices

The tax increase occurred at the wholesale level of the supply-chain and coincided with the monopoly’s announcement that the wholesale price of all cigarettes would increase by 6%. This increase in the wholesale price of cigarettes matches the change in the *ad valorem* excise rate from 5% to 11%, indicating that the new specific rate of ¥ 0.1/pack was absorbed into industry wholesale margins, rather than being passed along to the consumer. Note the weighted average wholesale price increases by more than 6% (Table 2), reflecting a change in the composition (i.e. market share) of cigarette sales by class over time.

**Table 2:** Cigarette pack prices and tax yields, China, 2014–2016

Variable	Cigarette class^a^						Weighted average
	I(A)	I(B)	II	III	IV	V	
**Average wholesale price, ¥/pack**							
2014	36.0	20.6	11.6	8.3	4.5	2.3	10.3
2015	38.2	21.8	12.3	8.8	4.8	2.4	11.2
2016	38.2	21.8	12.3	8.8	4.8	2.4	11.2
Change (%)	2.2 (6)	1.2 (6)	0.7 (6)	0.5 (6)	0.3 (6)	0.1 (6)	0.9 (9)
**Average retail price, ¥/pack**							
2014	43.0	23.0	13.0	9.5	5.0	2.5	11.6
2015	45.0	25.0	14.0	10.0	5.5	3.0	12.8
2016	45.0	25.0	14.0	10.0	5.5	3.0	12.8
Change (%)	2.0 (5)	2.0 (9)	1.0 (8)	0.5 (5)	0.5 (10)	0.5 (20)	1.2 (11)
**Average excise tax, ¥/pack**							
2014	13.5	8.1	4.8	2.4	1.3	0.7	3.6
2015	15.6	9.4	5.6	3.0	1.7	1.0	4.4
2016	15.6	9.4	5.6	3.0	1.7	1.0	4.4
Change (%)	2.1 (16)	1.3 (16)	0.8 (16)	0.6 (24)	0.4 (27)	0.2 (31)	0.8 (24)
**Average total tax,^b^ ¥/pack**							
2014	22.2	13.0	7.6	4.3	2.4	1.4	6.0
2015	24.9	14.7	8.6	5.0	2.9	1.7	7.1
2016	24.9	14.7	8.6	5.0	2.9	1.7	7.2
Change (%)	2.7 (12)	1.8 (13)	1.0 (13)	0.7 (17)	0.5 (20)	0.3 (25)	1.2 (19)

¥: yuan.

^a^ Classification according to the State Tobacco Monopoly Administration. Class I has a wide retail price span, so we divided class I in to (A) and (B), where I(A) spans retail prices above 43 ¥ per pack. However, given that I(A) has a very small market share, we don’t address this category in detail in this paper.

^b^ Total tax includes excise and other indirect taxes on consumption, but excludes enterprise income tax.

Notes: One cigarette pack contains 20 sticks. The conversion rate in 2016 was 1 United States dollars to  ¥ 6.64.

The impact of the tax increase on cigarette retail price varied across the classes, where the retail price of the cheapest class V brands increased by 20% (from ¥ 2.5 to ¥ 3.0) in nominal terms (Table 2). The increase for class V brands partly reflects the monopoly’s notice that retail margins should be at least 10% for all brands and so the increase in class V brand’s retail price of ¥ 0.5/pack included an increase in the retailers’ profit margin of ¥ 0.2/pack. We interpret this as an incentive for retailers to continue stocking cheaper brands in support of the monopoly’s optimization strategy. That is, cheaper brands are not as profitable as premium brands for retailers to stock, but the monopoly’s strategy includes the continued availability of cheap brands.

Mid-priced class III brands account for almost half of the market and the retail price of these key brands increased by 5% (from ¥ 9.5 to ¥ 10.0 in nominal terms; Table 2). The price gap between class III and more expensive brands widened marginally. In contrast, the price gap between class III and cheaper class brands narrowed, though not by as much as would have been the case, had the new specific excise of ¥ 0.10/pack also been passed-through, that is included in the price increase. Overall, the average retail price of cigarettes increased by 11% in nominal terms (from ¥ 11.6 to ¥ 12.8), or by 7% after accounting for inflation.

#### Production and sales

Table 3 shows the aggregate market outcomes, including the reported production and sales volumes by class, and total cigarette tax revenues as reported by the monopoly, with the class breakdown of revenues being estimated by the WHO tobacco tax simulation model. The monopoly reported that annual cigarette retail sales volumes decreased by 8%, from 127 to 117 billion packs between 2014 and 2016, representing 10 billion fewer packs sold annually. Most of the decrease occurred in 2016, with this being the first full calendar year after the May 2015 tax increase. The decreases in both 2015 and 2016 constitute the first annual contractions in demand since 2001.^24^

**Table 3:** Annual production, retail sales and tax revenues of cigarettes, China 2014–2016

Variable	Cigarette class						Total market
	I(A)	I(B)	II	III	IV	V	
**Production volume, billion packs**							
2014	2	24	13	58	24	8	129
2015	2	25	15	56	22	7	128
2016	2	23	15	52	20	6	118
Change (%)	0 (−9)	−1 (−4)	2 (14)	−6 (−11)	−4 (−16)	−2 (−22)	−11 (−9)
**Retail sales volume, billion packs**							
2014	2	24	13	57	23	8	127
2015	2	25	15	55	21	7	124
2016	2	23	15	52	20	6	117
Change (%)	0 (−8)	−1 (−3)	2 (15)	−6 (−10)	−4 (−15)	−2 (−21)	−10 (−8)
**Total tax revenue, billion ¥**							
2014	41	297	98	240	54	11	740
2015	44	345	120	262	58	11	840
2016	44	338	132	260	57	11	842
Change (%)	3 (7)	41 (14)	34 (35)	21 (9)	3 (5)	0 (2)	101 (14)

¥: yuan.

Notes: One cigarette pack contains 20 sticks. Total tax revenues include excise and other indirect taxes on consumption, but exclude enterprise income tax. The conversion rate in 2016 was 1 US$ to  ¥ 6.64.

Data source: State Tobacco Monopoly Administration.^24^^,^^25^

**Fig. 1:**
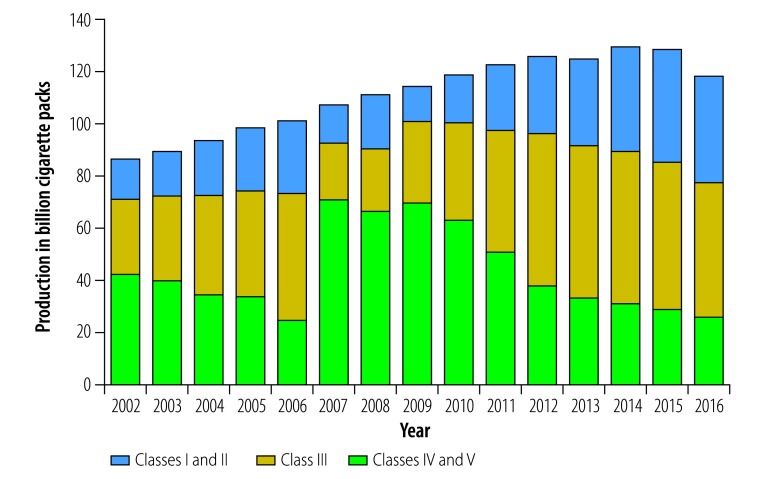
Annual cigarette production, China 2002–2016

Notes: Class I and II represent cigarette packs in the producer price range of > 7 yuan (¥)/pack, class III represents cigarette packs in the producer price range of 3–7 ¥/pack, and class IV and V represent cigarette packs in the producer price range of < 3 ¥/pack. The State Tobacco Monopoly Administration adjusted the price classification of cigarettes in 2007, leading to the unusual change in the proportion of classes.

Fig. 1 shows the pattern of cigarette production over the past decade, with the rapid growth in mid and high-priced brands reflecting the monopoly’s optimization strategy. Between 2014 and 2016, the volume of class I and II cigarettes continued to expand at a modest pace, while other classes decreased. The sales of low-priced class III to V brands decreased the most, suggesting it was smokers from lower socioeconomic groups that reduced or quit smoking the most. However, it is difficult to fully assess the impact even with this disaggregated data, because the changes by class will reflect a mix of consumer switching between classes, reduced conditional demand and reduced smoking prevalence.

Data source: State Tobacco Monopoly Administration.^24^

#### Tax incidence and revenue

Table 4 shows the tax incidence, that is, the burden of tax faced by consumers. Tax incidence is measured as the proportion of tax in the retail price of each class. The tax increase did not change the fundamental characteristics of tax incidence on cigarettes, with mid-price class III brands still recording the lowest tax incidence, due to being the highest-priced class included under the lower *ad valorem* tax rate of 36% at the producer level. Overall, the average total tax incidence rose from 51.7% to 55.7%.

**Table 4:** Changes in excise and total tax incidence per cigarette pack between 2014 and 2016, China

Tax	Tax incidence, %, by cigarette class^a^						Weighted average, %
	I(A)	I(B)	II	III	IV	V	
**Excise**							
2014	31.4	35.4	37.2	25.1	26.4	29.8	30.8
2015	34.7	37.6	39.9	29.5	30.4	32.4	34.4
2016	34.7	37.6	39.9	29.5	30.4	32.4	34.5
Change	3.4	2.3	2.8	4.5	4.1	2.6	3.7
**Total**							
2014	51.7	56.4	58.7	45.5	48.0	54.0	51.7
2015	55.4	58.8	61.7	50.4	52.2	56.0	55.6
2016	55.4	58.9	61.8	50.4	52.4	56.2	55.7
Change	3.8	2.5	3.0	4.9	4.4	2.2	4.0

¥: yuan.

^a^ Classification according to the State Tobacco Monopoly Administration. Class I has a wide retail price span, so we divided class I in to (A) and (B), where I(A) spans retail prices above 43 ¥ per pack. However, given that (A) has a very small market share, we don’t address this category in detail in this paper.

Notes: One cigarette pack contains 20 sticks. Total tax includes excise and other indirect taxes on consumption, but excludes enterprise income tax. The conversion rate in 2016 was 1 United States dollar to  ¥ 6.64.

Tax revenue from cigarettes reported by the monopoly amounted to ¥ 842 billion (US$ 127 billion) in 2016, representing almost 6.5% of fiscal revenue in China. Excise revenue from cigarettes amounted to about ¥ 520 billion, or around 62% of total cigarette tax revenue. Total tax revenues from cigarettes increased by 14%, from ¥ 740 to ¥ 842 billion between 2014 and 2016, reflecting an extra 101 billion yuan (US$ 15 billion) in tax revenues for the government. After accounting for inflation of around 3.5% over this period, cigarette tax revenues increased by 10% in real terms between 2014 and 2016.

#### Smoking prevalence

The Global Adult Tobacco Survey found that 27.7% of China’s adult population were smokers in 2015, representing about 318 million smokers.^10^ Assuming a price prevalence elasticity of −0.1 to−0.3 and using our estimated 7% of inflation-adjusted increase in cigarette prices, we predict that the reduction in the proportion of smokers could be between 0.2% and 0.6%. While this decrease may seem relatively modest, it would correspond to between 2.2 and 6.5 million fewer smokers. Thus, this tax increase would potentially make a measurable contribution to tobacco control efforts to reduce the number of smokers both in China and globally.

However, it is important to recognize that the single tax increase may not have such a significant or lasting impact on smoking prevalence in China. First, public expectations that cigarette prices will continue to increase may encourage more people to quit smoking, especially in countries where governments regularly increase tobacco taxes.^32^ However, taxes and prices in China have not changed for at least six-years before 2015, and therefore it seems unlikely that the single increase will have altered Chinese smokers’ expectations about future tax increases. Second, cigarettes in China remain very affordable, and will become more so over time due to continued growth in people’s incomes. Thus, the Chinese government may need to continue raising taxes and prices on cigarettes regularly to fully secure the estimated impact on smoking prevalence.

## Next steps

To confirm our estimated reduction in smoking prevalence due to the 2015 tax increase, field research is needed in China.

The Chinese government should raise tobacco taxes more significantly over the coming decade to pursue the country’s development objectives. In October 2016, President Xi Jinping announced the national strategy Healthy China 2030. The strategy sets several ambitious targets including to reduce smoking prevalence to 20% by 2030.^33^ To successfully achieve this target, policy-makers will need to implement a range of tobacco control policies. Evidence shows that taxation is the single most effective tobacco control measure and thus, higher tobacco taxes will be required to help achieve the Healthy China 2030 strategy.^33^ This link to broader development objectives in the SDGs and Healthy China 2030 can be facilitated through multisectoral research and dialogue, to develop consistent cross-sectoral objectives for tobacco tax policy.

In many countries, tobacco taxation is underutilized as a tobacco control measure.^29^ This is often due to industry interference in the policy-making process. In China, greater emphasis on health sector objectives in tobacco tax policy design could help against interference. Several other countries, such as the Philippines and Thailand, have also taken specific measures under Article 5.3 of the FCTC to protect policy-making from industry interference. The earmarking of some tobacco tax revenues to public health programmes could be another way to maximize benefits to the health sector, while also strengthening public support for further tax increases.^8^^,^^34^

Finally, China’s 2015 tax increase has highlighted the monopoly’s ability to under-shift taxes and to maintain a wide price gap among brands to support its long-term optimization strategy of promoting key mid- and high-price brands, while maintaining cheap offerings in the market. In particular, the availability of very cheap brands continues to pose a major challenge to public health, as it encourages smoking among young and poor people. The government may therefore need to develop tax policies tailored at raising the price of these cheap brands. Such policies could include significantly raising the specific rate, removing the *ad valorem* tiers at the producer level and/or implementing high minimum prices.

## Conclusion

Achieving the SDGs will require integrated, multisectoral approaches. Tobacco taxation is an example of such an approach, with progress needing to be underpinned by greater policy coherence between the government’s health and finance sectors. Indeed, it is well-documented that tobacco taxation policies contribute to improved health outcomes and better public finances, via reduced tobacco use yet increased tax revenue.^29^ China’s 2015 tobacco tax reform provides yet another practical demonstration of these dual benefits.
